# Diet Quality Shapes Metabolic and Immune Transcriptional Responses of Honey Bees Under Acute Heat Stress

**DOI:** 10.3390/insects17080780

**Published:** 2026-07-28

**Authors:** Hyunjee Kim, Olga Frunze, Sang Mi Han, Soon Ok Woo, Jewon Jung, Hyung-Wook Kwon

**Affiliations:** 1 Department of Life Sciences and Convergence Research Center for Insect Vectors (CRCIV), Incheon National University R&D Complex, 265 Harmony-ro, Yeonsu-gu, Incheon 22014, Republic of Korea; beamed79@hanmail.net (H.K.); frunzeo1@inu.ac.kr (O.F.); 2 Apiculture Division, Department of Agricultural Biology, National Institute of Agricultural Sciences, Rural Development Administration (RDA), Wanju 55365, Republic of Korea; sangmih@korea.kr (S.M.H.); wooso1@korea.kr (S.O.W.); 3 Department of SmartBio, College of Life and Health Science, Kyungsung University, Busan 48434, Republic of Korea; jjewony@gmail.com

**Keywords:** honey bee, heat stress, nutrition, pollen substitute, gene expression, climate change

## Abstract

Climate warming increasingly threatens honey bee health, yet diet-associated molecular patterns under thermal stress remain poorly understood. In this study, newly emerged honey bees were fed eight dietary treatments, including natural diets (honey and bee bread) and artificial diets (sugar syrup and pollen substitute diets), for 5 days before exposure to acute heat stress (45 °C for 4 h). Transcriptional responses were quantified by quantitative real-time PCR (qRT-PCR) using genes associated with nutrient sensing, metabolism, immunity, and stress responses, and the resulting expression profiles were analyzed using principal component analysis, hierarchical clustering, and univariate statistical analyses. Distinct diet-associated transcriptional patterns were observed under both baseline and heat-stress conditions. Under baseline conditions, transcriptional variation was primarily associated with nutrient-sensing and metabolic pathways, whereas under heat-stress conditions, metabolic and stress-response pathways contributed more strongly to transcriptional variation. Sugar syrup-fed honey bees exhibited elevated expression of several immune-related genes following heat stress, suggesting greater physiological disturbance. In contrast, honey- and bee bread-based diets exhibited more coordinated regulation of nutrient-sensing and metabolic pathways and comparatively lower expression of selected immune-related genes. These findings demonstrate that dietary quality is associated with distinct molecular profiles under thermal stress and provide mechanistic insights into the relationship between nutrition and gene expression under heat-stress conditions, supporting the development of nutrition-based strategies for promoting honey bee health under climate warming.

## Simple Summary

Honey bees are essential pollinators that support food production and natural ecosystems. However, rising temperatures associated with climate change are placing increasing physiological stress on honey bee populations worldwide. Nutrition is known to influence honey bee health, yet diet-associated molecular patterns under heat stress remain poorly understood. In this study, we compared the effects of natural diets, including honey and bee bread, with artificial diets, including sugar syrup and pollen substitute diets, on the expression of metabolic and immune-related genes before and after acute heat exposure. We found distinct diet-associated transcriptional patterns under both baseline and heat-stress conditions. Honey bees fed nutritionally complex diets exhibited more coordinated regulation of metabolic and stress-response pathways, whereas honey bees fed sugar syrup showed stronger activation of immune-related genes under heat-stress conditions, suggesting greater physiological disturbance. These findings demonstrate that dietary quality is associated with distinct molecular profiles under thermal stress and suggest that nutritionally complex diets may support honey bee physiological regulation during heat exposure, thereby supporting sustainable pollination services and agricultural production.

## 1. Introduction

Honey bees (*Apis mellifera* L.) are key agricultural pollinators that support global food production, biodiversity, and ecosystem stability, while also providing valuable hive products such as honey, pollen, and royal jelly [1]. Despite their ecological and economic importance, honey bee colonies have experienced substantial global losses in recent decades, raising concern for food security and ecosystem functioning [2]. These declines are driven by multiple interacting stressors, including climate change, nutritional deficiencies, pathogens, pesticides, habitat degradation, and beekeeping practices [3,4,5]. Among these, climate warming has emerged as an increasingly important threat to pollinator health [6].

Honey bees regulate brood nest temperature through collective behaviors such as fanning and endothermic activity of the flight muscles [7]. However, elevated temperatures increase metabolic demands and disrupt physiological homeostasis, impairing immune function, development, and foraging performance [7,8,9,10].

Climate change disrupts nutritional landscapes by altering flowering phenology, nectar, and pollen production, potentially creating phenological mismatches between bees and floral resources [11]. Adequate nutrition is essential for colony performance and resilience, influencing longevity, immune competence, and overwintering success [12,13,14]. Despite their frequent co-occurrence under climate change, nutritional and thermal stress have largely been investigated independently [15].

Honey bees obtain carbohydrates primarily from nectar (processed into honey), which provides energy for flight and thermoregulation, whereas pollen provides proteins, lipids, vitamins, and minerals essential for growth, immunity, and colony development [14,16,17,18]. During periods of pollen scarcity, beekeepers commonly provide artificial pollen substitutes to support colony health [19].

At the molecular level, nutrition and temperature influence interconnected metabolic, endocrine, and immune pathways [20,21]. Insulin-like peptides (ILPs), TOR signaling, juvenile hormone (JH), and vitellogenin (vg) regulate nutrient sensing, metabolism, development, and longevity [22,23,24]. Honey bees also rely on innate immune pathways, including the Immune Deficiency (Imd) pathway, in which the transcription factor *Relish* activates antimicrobial peptides such as *defensin2* to protect against microbial infection [25,26].

Although nutrition and thermal stress have been widely studied separately, diet-associated metabolic and immune regulation under acute heat-stress conditions remains poorly understood. This knowledge gap is particularly important under climate change, where increasingly frequent short-term heat events may challenge honey bee physiology. To address this gap, we investigated transcriptional profiles associated with short-term dietary treatments before and after subsequent acute heat stress in honey bees using an integrated framework of multivariate and univariate analyses. Specifically, we aimed to characterize diet-dependent transcriptional profiles under baseline conditions, characterize these profiles under acute heat stress, and identify metabolic and immune genes that contribute most strongly to dietary group separation. This study provides mechanistic insights into the association between nutrition and gene expression under thermal stress and highlights the potential importance of nutritional status in supporting honey bee health under climate change.

## 2. Materials and Methods

### 2.1. Diet Preparation

#### 2.1.1. Carbohydrate and Natural Diets

Three carbohydrate-based diets were included for comparison: 30% sugar syrup (Ssyrup), sugar-fed honey (Shoney), and acacia honey (Ahoney). Ssyrup served as a carbohydrate-only reference diet [27]. Shoney was a commercially available sugar-fed honey produced by Sannaedeul Farm Co., Ltd. (Gimhae, Gyeongsangnam-do, Republic of Korea), whereas Ahoney was certified Korean acacia honey derived from *Robinia pseudoacacia* that met the quality standards of the Korean Food Code.

Bee bread (Bbread) collected from three healthy colonies maintained at the experimental apiary was used as a natural diet. A positive control treatment (Cont) consisted of bee bread and 30% sugar syrup provided simultaneously in separate feeding tubes, allowing honey bees to consume both food sources ad libitum. Because this treatment supplied both natural pollen-derived nutrients and carbohydrates, it served as a nutritionally balanced reference diet for comparison with the experimental diets. This feeding regime was intended to approximate the simultaneous availability of pollen-derived nutrients and carbohydrates within a colony and was used as the reference feeding strategy for the experimental design.

All diets were stored in airtight containers at 4 °C until use.

#### 2.1.2. Artificial Protein-Containing Diets

For comparison under subsequent heat-stress conditions, three artificial pollen substitute diets, Diet1(S), Diet2(M), and Diet3(O), were prepared based on a previously developed pollen substitute formulation [19,28]. All diets shared the same basal ingredients, while selected functional additives differed among diets (Table S1). The basal formulation contained inosine-5′-monophosphate (IMP) and guanosine-5′-monophosphate (GMP) (Sigma-Aldrich, Milwaukee, St. Louis, MO, USA) to improve diet palatability and voluntary consumption [29]. Depending on the diet formulation, apple juice, chlorella powder, Soytide (CJ Global Food and Bio Company, Seoul, Republic of Korea), or microbiome-fermented defatted soybean powder produced using *Bacillus subtilis* (M) was incorporated as a functional ingredient. Apple juice and chlorella were included as nutritional supplements providing carbohydrates and bioactive compounds, whereas the fermented soybean products served as highly digestible protein sources with reported enzymatic and antimicrobial activities [30].

### 2.2. Cage Experiment and Sampling

Cage experiments were conducted for 5 days in an incubator maintained at 33 ± 2 °C and 55 ± 5% relative humidity. A 5-day feeding period was selected to encompass the early adult maturation phase, during which newly emerged worker bees undergo rapid diet-dependent physiological and metabolic changes detectable at the transcriptional level [19,28]. Three healthy honey bee colonies maintained at the Incheon National University Apiary were used in this study. Colonies were selected based on comparable colony strength and the absence of visible signs of disease or parasite infestation at the time of sampling. One capped brood frame was collected from each colony and maintained in an incubator until adult emergence.

Newly emerged worker bees (<24 h old) were randomly allocated to experimental cages (17 × 14.5 × 9.5 cm), with 30 honey bees per cage. Eight dietary treatments were evaluated, with three independent cages per treatment (24 cages in total). Each cage was considered one biological replicate throughout the experiment and served as the experimental unit for all statistical analyses. Experimental diets were provided ad libitum throughout the 5-day feeding period. Water was supplied continuously using a 15 mL feeding tube filled with boiled and cooled water. The tube cap was perforated with a fine pin to regulate water flow and positioned upside down on top of each cage.

**Figure 1:**
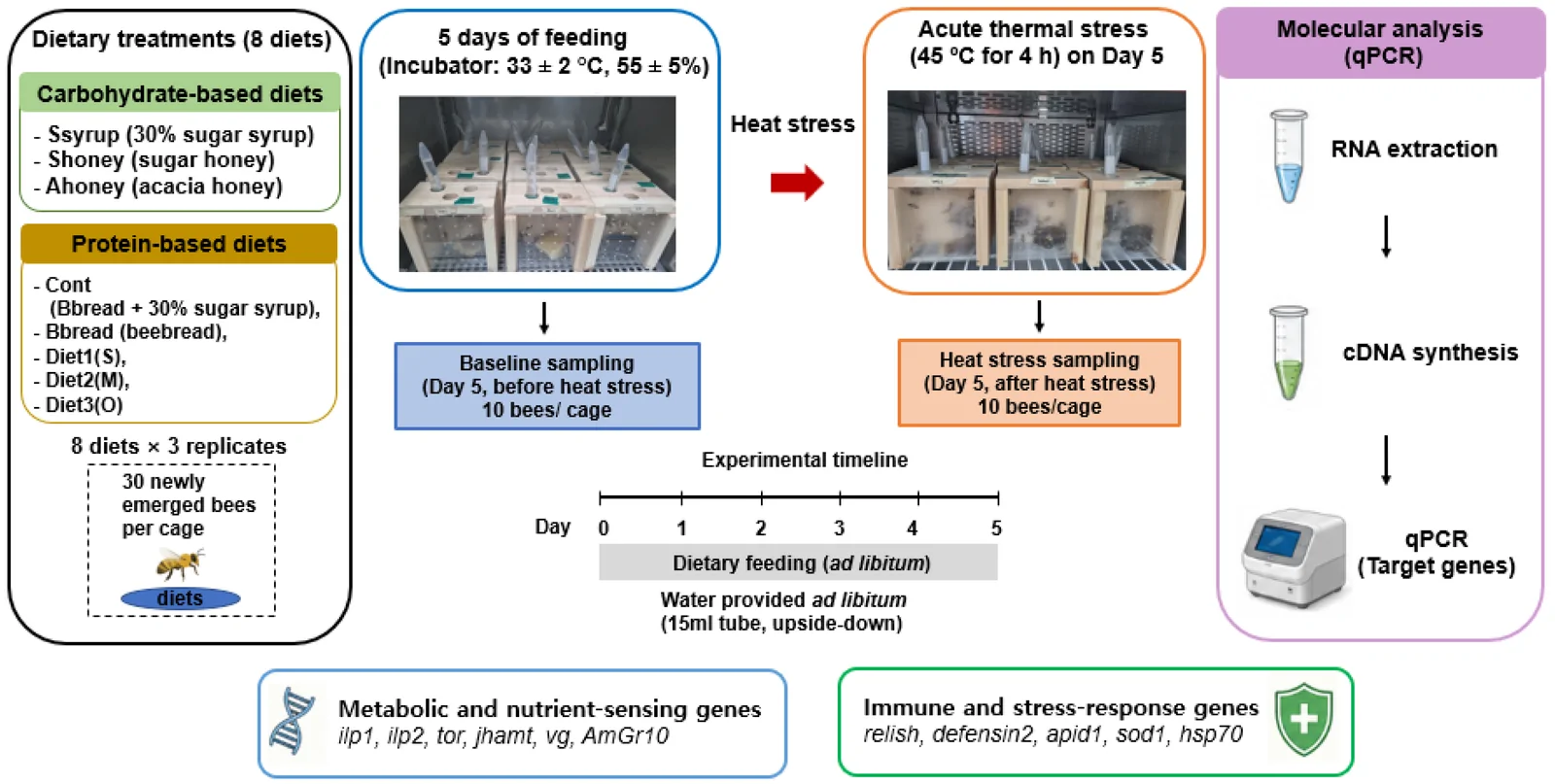
Schematic overview of the experimental design. Eight dietary treatments were tested in a cage experiment (*n* = 3 cages per treatment; each cage represented one biological replicate), with 30 newly emerged worker honey bees per cage. Honey bees were fed ad libitum for 5 days under controlled conditions (33 ± 2 °C, 55 ± 5% RH). On Day 5, ten worker bees were randomly collected from each cage under baseline conditions for gene expression analysis. For each biological replicate, pooled head samples (three worker heads) and pooled whole-body samples (two intact worker bees) were prepared for qRT-PCR analysis. The remaining 20 bees were exposed to acute heat stress (45 °C for 4 h), after which an additional ten worker bees were collected and processed using the same sampling procedure.

On Day 5, ten worker bees were randomly collected from each cage under baseline conditions for gene expression analysis. The remaining 20 honey bees were immediately transferred to a temperature-controlled incubator and exposed to acute heat stress (45 °C for 4 h) following a previously described protocol [31]. Following heat exposure, an additional ten worker bees were randomly collected from each cage for gene expression analysis. The overall experimental design is illustrated in Figure 1.

### 2.3. RNA Extraction and cDNA Synthesis

Because each cage represented one biological replicate, three independent biological replicates were analyzed for each dietary treatment (*n* = 3 cages per treatment). At each sampling point, ten worker honey bees were randomly collected from each cage, immediately placed into 15 mL conical tubes, snap-frozen on dry ice, and stored at −80 °C until further processing.

For qRT-PCR analysis, 5 honey bees were randomly selected from each biological replicate (cage). Under frozen conditions, the heads of three honey bees were dissected on dry ice and pooled to generate one head sample, whereas two additional intact honey bees were pooled to generate one whole-body sample. Thus, each dietary treatment included three biological replicates for head tissues and three biological replicates for whole-body tissues, with each biological replicate represented by one pooled sample obtained from a single cage.

Head tissues were used to quantify the expression of *jhamt*, *tor*, *ilp1*, *ilp2*, *hsp70*, and *AmGr10*, whereas whole-body samples were used to measure *vg*, *relish*, *defensin2*, *apid1*, and *sod1* [32]. These genes were selected to assess pathways involved in metabolic regulation (*jhamt*, *tor*, *ilp1*, *ilp2*, *AmGr10*, and *vg*) and immune or stress responses (*hsp70*, *relish*, *defensin2*, *apid1*, and *sod1*). Expression of juvenile hormone acid methyltransferase (*jhamt*), a key enzyme in juvenile hormone (JH) biosynthesis, was used as a molecular proxy for potential JH biosynthetic activity [33].

Total RNA was extracted from pooled head and whole-body samples using the RNeasy Mini Kit (Qiagen, Valencia, CA, USA) according to the manufacturer’s instructions. RNA concentration and purity were assessed spectrophotometrically, and only RNA samples with A260/A280 ratios between 1.8 and 2.0 were used for downstream analysis. Complementary DNA (cDNA) was synthesized using the RNA to cDNA EcoDry™ Premix (Oligo dT) kit (Takara Bio USA, Inc., San Jose, CA, USA) according to the manufacturer’s instructions. Each reverse transcription reaction contained 1 μg of total RNA in a final volume of 20 μL adjusted with RNase-free water. Reverse transcription was performed at 42 °C for 60 min, followed by enzyme inactivation at 70 °C for 10 min.

### 2.4. Quantitative Real-Time PCR (qRT-PCR)

Relative expression levels of the target genes were quantified by quantitative real-time PCR (qRT-PCR). Each reaction was performed in a total volume of 20 μL containing 2 μL of cDNA template, 10 μL of Brilliant III Ultra-Fast SYBR Green QPCR Master Mix (Agilent Technologies, Cedar Creek, TX, USA), gene-specific forward and reverse primers (Table S2), and nuclease-free water (AM9930, Invitrogen, Life Technologies Corporation, Austin, TX, USA). Amplification was carried out using an AriaMx Real-Time PCR System (Agilent Technologies, Santa Clara, CA, USA) under the following cycling conditions: initial denaturation at 95 °C for 5 min, followed by 40 cycles of 95 °C for 30 s, 60 °C for 30 s, and 72 °C for 30 s. Melting curve analysis was performed following amplification to confirm the specificity of each PCR product.

Relative gene expression levels were calculated using the 2^−ΔΔCt^ method with *β-actin* as the internal reference gene [27]. All primer pairs used in this study were adopted from previously published and validated studies (Table S2). Melting curve analysis confirmed the specificity of all amplification products. Each pooled biological sample (one pooled sample per cage) was analyzed in three technical replicates, and the mean Ct value was used for subsequent statistical analyses.

### 2.5. Statistical Analysis

**Figure 2:**
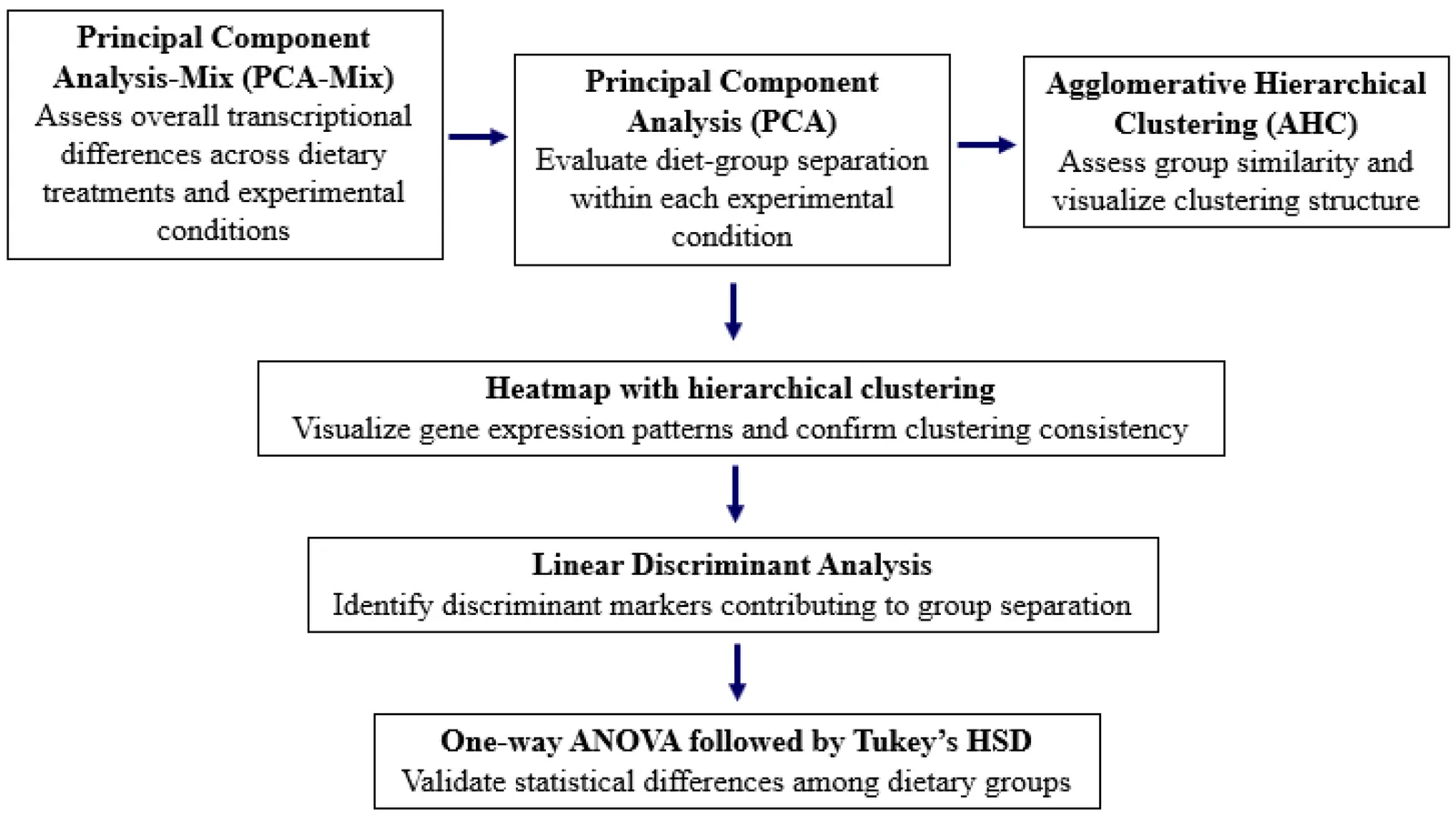
Workflow of multivariate and univariate statistical analyses and data visualization used to evaluate dietary differences and identify discriminant markers.

All statistical analyses were performed using Microsoft Excel and XLSTAT (Addinsoft, Pearson Edition 2014, Paris, France) following the workflow shown in Figure 2. The cage was considered the experimental unit for all analyses. To explore overall treatment-related patterns, principal component analysis for mixed data (PCA-Mix) was first applied to evaluate global transcriptional profiles across dietary treatments under both the 5-day feeding and heat-stress conditions. Principal component analysis (PCA) was then conducted separately within each condition to assess diet-dependent separation. Agglomerative hierarchical clustering (AHC) was used to evaluate group similarity and visualize clustering structure based on Euclidean distance and Ward’s linkage method.

In addition, heatmap analysis with hierarchical clustering was performed to visualize relative gene expression patterns across dietary treatments and to complement the multivariate results obtained from PCA and AHC. Gene expression values were standardized (z-score transformation) before visualization, and clustering was applied to both genes and samples to identify co-expression patterns and treatment-related clustering.

To identify variables contributing most strongly to group separation, linear discriminant analysis (LDA) was conducted based on the multivariate structure revealed by PCA and AHC. Variables with the highest standardized canonical coefficients were considered potential discriminative markers.

For univariate analysis, gene expression values were summarized at the cage level. Normality and homogeneity of variance were assessed using the Shapiro–Wilk and Levene’s tests, respectively. Selected discriminative markers were further evaluated using one-way analysis of variance (ANOVA), followed by Tukey’s honestly significant difference (HSD) post hoc test. Statistical significance was defined as *p* < 0.05. The analyses evaluated dietary differences separately under baseline and heat-stress conditions and did not formally test changes between conditions within diets or the diet × heat stress interaction.

## 3. Results

### 3.1. PCA-Mix Analysis of Gene Expression Profiles Following 5-Day Dietary Treatment and Heat Stress

**Figure 3:**
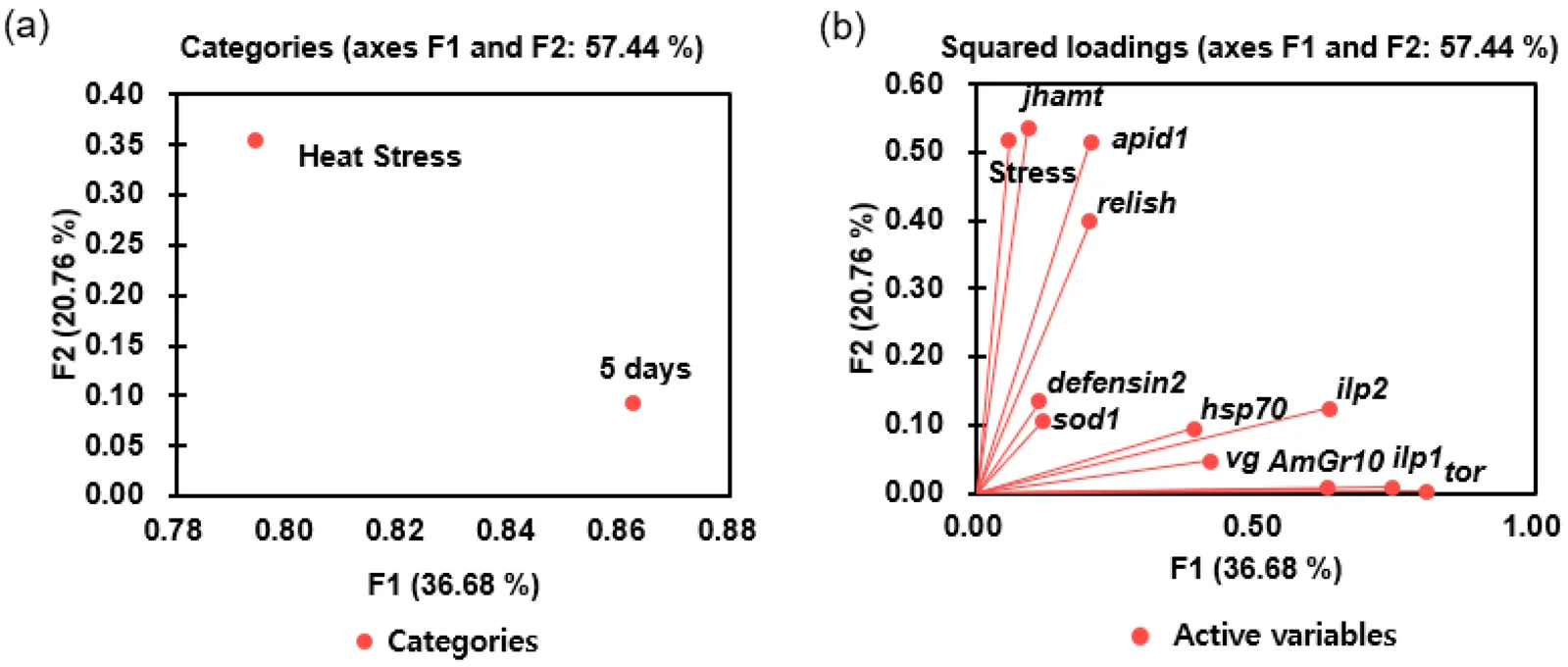
PCA-Mix analysis of gene expression profiles under 5-day feeding and heat-stress conditions. (**a**) Score plot showing separation between the 5-day feeding and heat-stress groups along the first two principal components (F1 and F2), explaining 57.44% of the total variance. (**b**) Loading plot showing the contributions of metabolic- and immune/stress-related genes to the first two principal components.

PCA-Mix analysis showed distinct distributions of samples from the 5-day feeding and heat-stress conditions along the first two principal components, which together explained 57.44% of the total variance (Figure 3). The first principal component (F1, 36.68%) was characterized by high positive loadings for genes associated with metabolic regulation and nutrient signaling, including *tor* (0.805), *ilp1* (0.744), *ilp2* (0.631), *AmGr10* (0.628), and *vg* (0.418). In contrast, the second principal component (F2, 20.76%) showed higher loadings for genes related to immune and stress-associated pathways, such as *jhamt* (0.537), *apid1* (0.516), and *relish* (0.399). Together, these findings indicate that transcriptional variation observed across dietary treatments and experimental conditions was organized along two principal axes: a metabolic regulatory axis (F1) and an immune/stress-response axis (F2).

### 3.2. Multivariate Analysis of Gene Expression Responses to 5-Day Dietary Treatments

**Figure 4:**
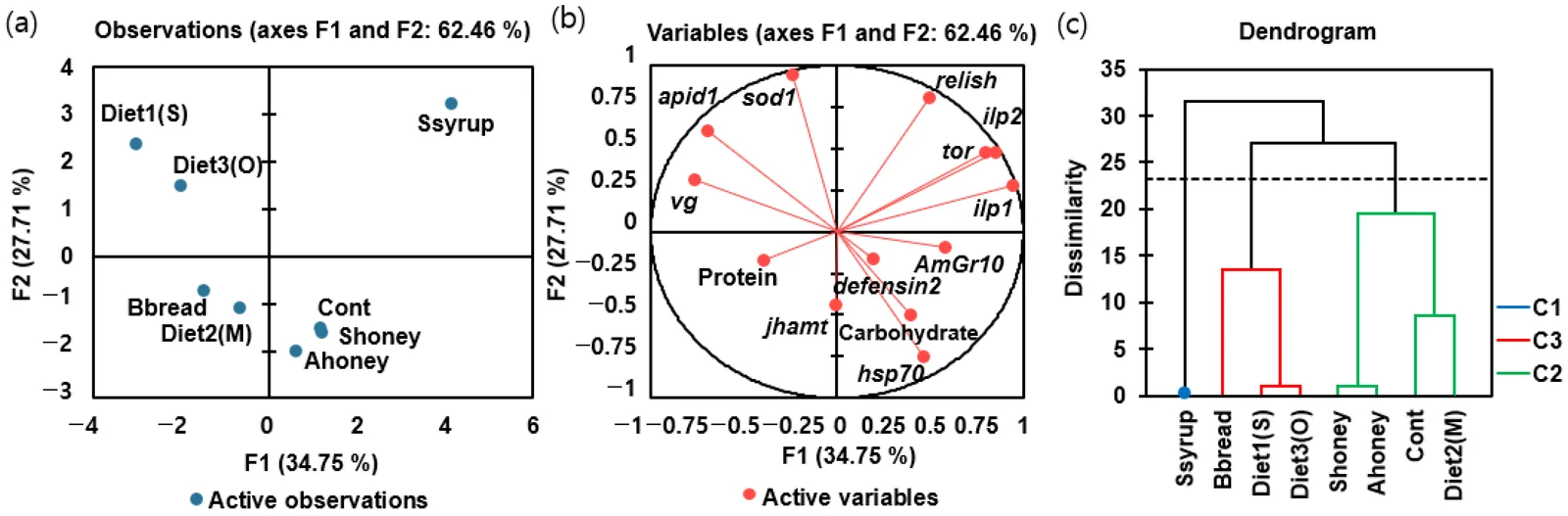
PCA of gene expression profiles following 5-day dietary treatments. (**a**) PCA score plot showing the distribution of dietary groups along the first two principal components (F1 = 34.75%, F2 = 27.71%). (**b**) PCA loading plot illustrating the contribution of gene variables to dietary separation. (**c**) AHC dendrogram based on Euclidean distance and Ward’s method showing three major dietary clusters after 5 days of feeding. The horizontal dashed line indicates the dissimilarity threshold used to define the three major clusters.

PCA was performed to evaluate the effects of 5-day dietary treatments on global gene expression (Figure 4a). The first two principal components (F1, 34.75% and F2, 27.71%) together explained 62.46% of the total variance.

The PCA score plot revealed clear diet-dependent separation of the eight dietary treatments. Ssyrup was positioned on the positive side of F1, whereas Diet1(S) and Diet3(O) clustered closely on the negative side of F1. Ahoney, Shoney, and Cont were located near the center of the plot, while Diet2(M) occupied an intermediate position close to Bbread, indicating a transcriptional profile more similar to those of the natural protein-containing diets.

Along F2, most artificial diets (Diet1(S), Diet3(O), and Ssyrup) were distributed toward the positive direction, whereas the natural diets were generally positioned toward the negative direction, with Diet2(M) representing an exception.

The PCA loading plot (Figure 4b) showed that nutrient-sensing and metabolic genes, including *ilp1*, *ilp2*, and *tor*, contributed primarily to the separation along F1, whereas *vg* showed a strong negative loading on the same axis (Table S3). In contrast, immune-related genes, particularly *relish* and *sod1*, contributed more strongly to F2, indicating that the second principal component reflected immune-related transcriptional variation.

AHC analysis further supported the PCA results, identifying three distinct dietary clusters: Cluster 1 (Ssyrup), Cluster 2 (Shoney, Ahoney, Cont, and Diet2(M)), and Cluster 3 (Bbread, Diet1(S), and Diet3(O)) (Figure 4c).

These results characterize the baseline transcriptional profiles established after 5 days of dietary treatment and provide a reference for subsequent analyses under heat-stress conditions.

### 3.3. Dietary Effects on Gene Expression Profiles Under Heat-Stress Conditions

**Figure 5:**
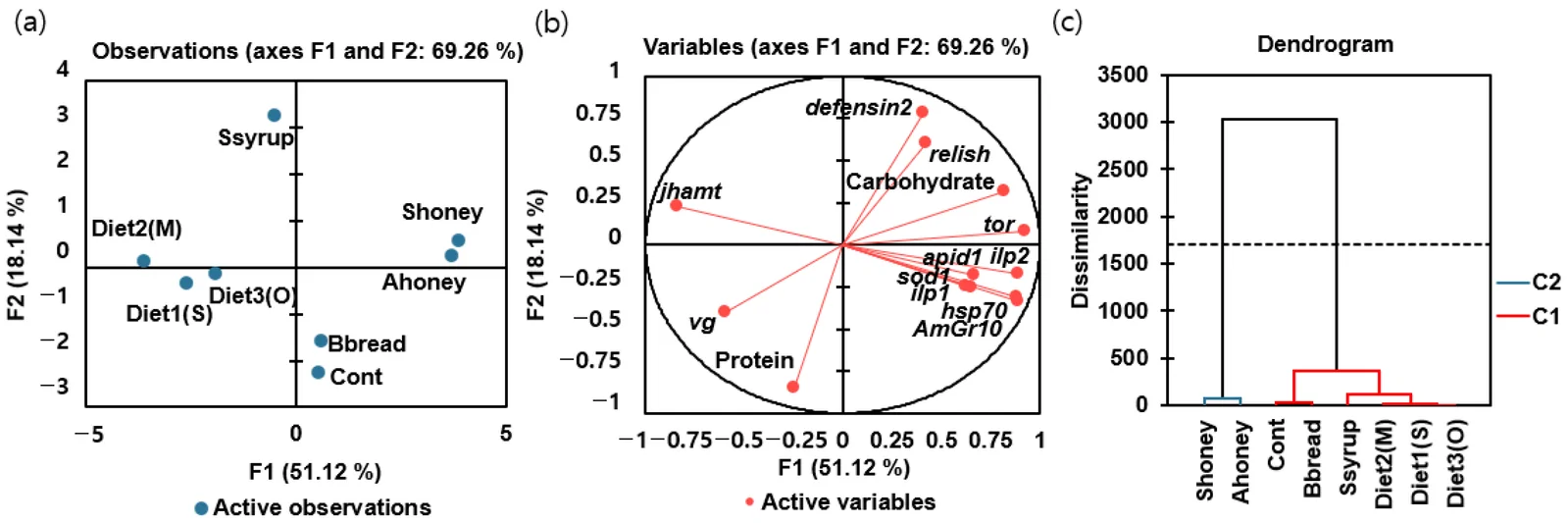
PCA of dietary effects under heat-stress conditions. (**a**) PCA score plot showing the distribution of dietary groups along the first two principal components (F1 = 51.12%, F2 = 18.14%). (**b**) PCA loading plot illustrating the contribution of gene variables to dietary separation. (**c**) AHC dendrogram based on Euclidean distance and Ward’s method showing the clustering pattern of diets under heat-stress conditions. The horizontal dashed line indicates the dissimilarity threshold used to define the two major clusters.

To characterize diet-associated transcriptional profiles under thermal challenge, PCA was performed on gene expression profiles under heat-stress conditions (Figure 5a). The first two principal components (F1, 51.12% and F2, 18.14%) explained 69.26% of the total variance in gene expression. The PCA score plot revealed clear dietary separation primarily along F1. Among the carbohydrate-based diets, Ssyrup was positioned on the negative side of F1, whereas Shoney and Ahoney were located on the positive side, indicating distinct transcriptional profiles among the carbohydrate-based diets under heat-stress conditions.

In contrast, the three pollen substitute diets (Diet1(S), Diet2(M), and Diet3(O)) clustered closely together and were positioned on the negative side of F1, whereas the natural protein-containing diets (Bbread and Cont) were located on the positive side of F1. This distribution indicates that the first principal component largely separated diets according to their nutritional origin.

Along the second principal component (F2), dietary treatments showed additional separation patterns. Ssyrup was positioned toward the positive direction of F2, whereas Bbread and Cont were located toward the negative direction, with the remaining diets occupying intermediate positions.

The loading plot (Figure 5b) provided further insight into the genes contributing to these separations. Nutrient-sensing and metabolic regulatory genes, including *tor*, *AmGr10*, and *ilp2*, showed strong positive loadings on F1 (Table S3), indicating that this axis primarily reflects diet-dependent metabolic regulation. In contrast, *jhamt* showed a strong negative loading on F1, suggesting an opposing transcriptional pattern relative to the insulin/TOR-associated genes. In addition, *hsp70* contributed substantially to F1, reflecting transcriptional responses associated with heat stress. Several immune-related genes, including *relish* and *defensin2*, contributed more strongly to F2, supporting the interpretation that the second axis reflects immune-related transcriptional variation.

To further evaluate the PCA-based separation, AHC was performed (Figure 5c). The resulting dendrogram identified two major dietary clusters: Cluster 1 (Cont, Bbread, Ssyrup, Diet2(M), Diet1(S), and Diet3(O)) and Cluster 2 (Shoney and Ahoney).

### 3.4. Linear Discriminant Analysis of Nutrition- and Immune-Related Gene Expression

**Figure 6:**
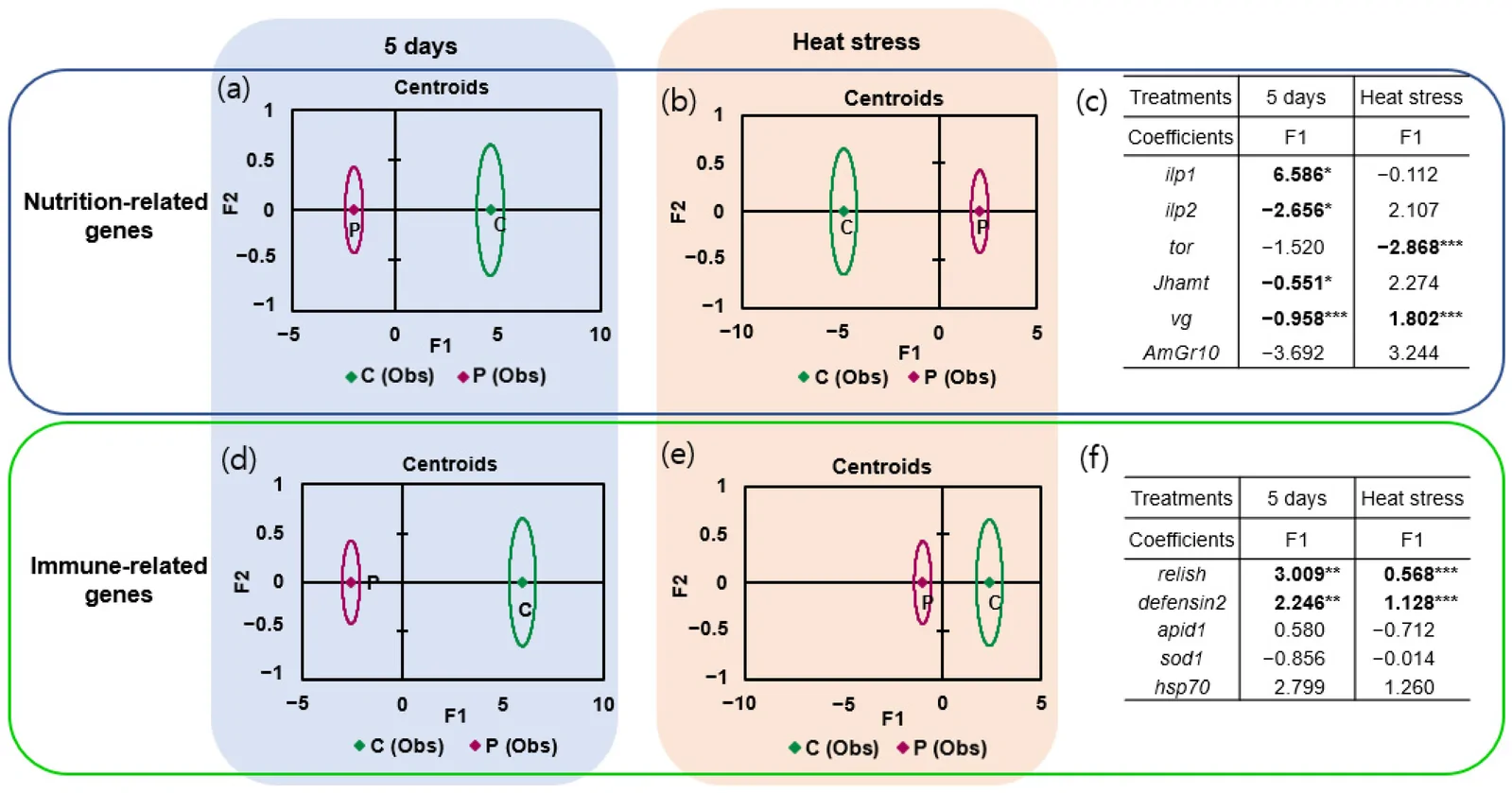
Linear discriminant analysis (LDA) of nutrition- and immune-related gene expression under 5-day feeding and heat-stress conditions. (**a**–**c**) LDA based on nutrition-related genes after 5 days of feeding (**a**) and under heat stress (**b**), with the standardized canonical discriminant function coefficients for F1 shown in (**c**). (**d**–**f**) LDA based on immune-related genes after 5 days of feeding (**d**) and under heat stress (**e**), with standardized canonical discriminant function coefficients for F1 shown in (**f**). In centroid plots, C represents carbohydrate-based diets (Ssyrup, Shoney, and Ahoney), and P represents protein-containing diets (Diet1(S), Diet2(M), Diet3(O), Cont, and Bbread). Ellipses indicate 95% confidence regions around group centroids. Bold values indicate genes showing significant between-group differences based on the unidimensional test of equality of class means, with asterisks denoting significance levels (* *p* < 0.05, ** *p* < 0.01, *** *p* < 0.001). Ssyrup was classified within the carbohydrate category based on its macronutrient composition.

LDA was conducted to evaluate group-level differences between carbohydrate-based diets (C) and protein-containing diets (P) under both the 5-day feeding and heat-stress conditions (Figure 6).

After 5 days of feeding, significant separation between C and P diets was detected for nutrition-related genes (Pillai’s trace = 0.908, F(6, 23) = 37.995, *p* < 0.001) and immune-related genes (Pillai’s trace = 0.942, F(5, 24) = 78.405, *p* < 0.001). Under heat-stress conditions, dietary group discrimination was significant for both nutrition-related genes (Pillai’s trace = 0.915, F(6, 23) = 41.250, *p* < 0.001) and immune-related genes (Pillai’s trace = 0.719, F(5, 24) = 12.285, *p* < 0.001). Overall, multivariate tests indicated significant discrimination between carbohydrate- and protein-based dietary groups under both physiological conditions.

Centroid plots based on nutrition-related genes showed clear separation between C and P groups along the first discriminant function (F1) after 5 days of feeding (Figure 6a), indicating diet-dependent differences in metabolic gene expression under baseline conditions. Under heat-stress conditions, separation between dietary groups was also evident, with slightly greater group dispersion (Figure 6b).

Standardized canonical discriminant function coefficients identified the genes contributing most strongly to dietary discrimination (Figure 6c). After 5 days of feeding, *ilp1*, *ilp2*, *vg*, and *jhamt* showed the strongest contributions to F1. Under heat-stress conditions, however, *tor* and *vg* contributed more strongly, indicating a shift in the metabolic genes contributing to dietary separation. *AmGr10* also showed relatively strong standardized coefficients, although its expression did not differ significantly between dietary groups.

LDA based on immune-related genes also showed separation between C and P groups after 5 days of feeding (Figure 6d). Under heat-stress conditions, separation remained detectable but was less pronounced than that observed after 5 days of feeding (Figure 6e). Standardized canonical discriminant function coefficients indicated that *relish* and *defensin2* contributed to discrimination between dietary groups, with stronger contributions observed after 5 days of feeding than under heat stress (Figure 6f). These results indicate that dietary group discrimination was consistently detectable under both physiological conditions, although the genes contributing most strongly to separation differed between baseline and heat-stress conditions.

### 3.5. Diet-Dependent Expression of Key Metabolic and Immune Genes Under Feeding and Heat-Stress Conditions

**Figure 7:**
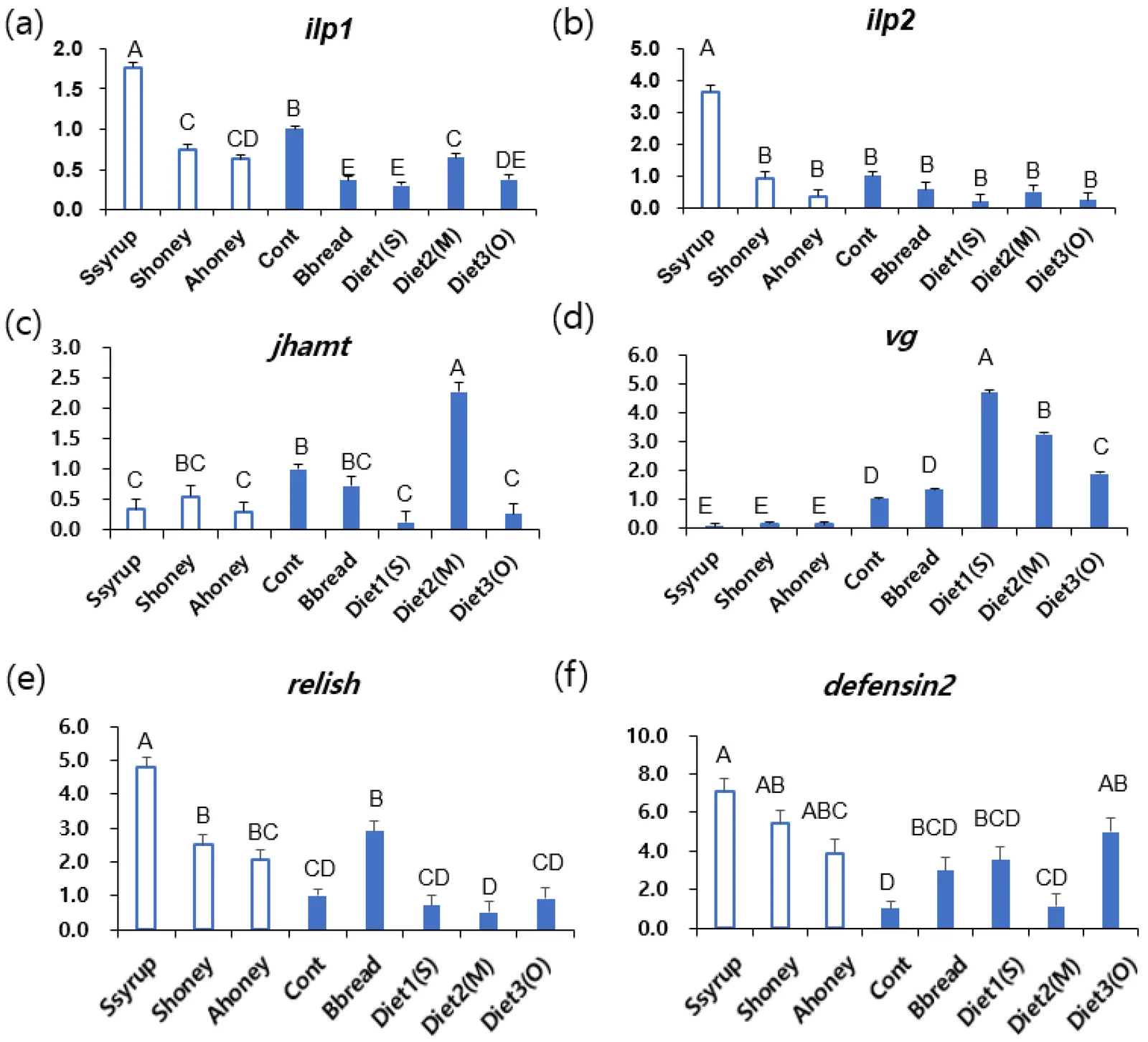
Diet-dependent expression of key metabolic and immune-related genes following 5-day feeding and heat-stress exposure. (**a**–**d**) Relative expression levels of metabolic regulatory genes (*ilp1*, *ilp2*, *jhamt*, and *vg*) after 5 days of dietary treatment. (**e**,**f**) Relative expression levels of immune-related genes (*relish* and *defensin2*) following acute heat-stress exposure (45 °C for 4 h). Bars represent mean ± SE. Different uppercase letters indicate significant differences among dietary treatments (one-way ANOVA followed by Tukey’s post hoc test, *p* < 0.05). Open bars represent carbohydrate-based diets (Ssyrup, Shoney, and Ahoney), whereas filled bars represent protein-containing diets (Cont, Bbread, Diet1(S), Diet2(M), and Diet3(O)).

To further evaluate the genes identified as major contributors to dietary group separation in the discriminant analysis (Figure 6), univariate analyses were performed for selected metabolic and immune-related genes (Figure 7).

After 5 days of dietary treatment, genes involved in insulin signaling and juvenile hormone pathways exhibited pronounced diet-dependent expression patterns (Figure 7a–d). Expression levels of *ilp1* (F(7, 22) = 80.265, *p* < 0.001) and *ilp2* (F(7, 22) = 28.586, *p* < 0.001) were significantly higher in the Ssyrup group than in all other dietary treatments (Figure 7a,b). *jhamt* expression also differed significantly among diets (F(7, 22) = 17.983, *p* < 0.001), with the highest expression observed in Diet2(M) and generally higher expression in protein-containing diets (Cont, Bbread, Diet1(S), Diet2(M), and Diet3(O)) than in carbohydrate-based diets (Ssyrup, Shoney, and Ahoney) (Figure 7c). In contrast, *vg* expression was strongly dependent on protein availability (F(7, 22) = 522.699, *p* < 0.001), showing markedly elevated levels in protein-containing diets (Cont, Bbread, Diet1(S), Diet2(M), Diet3(O)) and minimal expression in carbohydrate-based diets (Ssyrup, Shoney, and Ahoney) (Figure 7d).

Under heat-stress conditions, immune-related gene expression differed among dietary treatments (Figure 7e,f). *relish* expression differed significantly among diets (F(7, 22) = 22.758, *p* < 0.001), with the highest levels observed in the Ssyrup group (Figure 7e). *defensin2* showed a similar pattern (F(7, 22) = 12.831, *p* < 0.001), displaying elevated expression in the Ssyrup group and comparatively lower levels in most protein-containing diets (Figure 7f).

Overall, both *relish* and *defensin2* showed higher expression in carbohydrate-based diets (Ssyrup, Shoney, and Ahoney) than in protein-containing diets (Cont, Bbread, Diet1(S), Diet2(M), Diet3(O)). These results indicate that diet-associated differences remained evident under acute heat-stress conditions, particularly for immune-related genes. These univariate results are consistent with the patterns observed in the heatmap and further support clear diet-dependent differences in gene expression (Figure S1).

## 4. Discussion

This study shows that short-term dietary treatment is associated with distinct metabolic and immune gene expression profiles under both baseline and acute heat-stress conditions. These findings highlight the importance of considering dietary background when interpreting transcriptional patterns observed under acute heat stress.

### 4.1. Baseline Dietary Structuring After 5-Day Feeding

Under non-stressed conditions, dietary composition primarily influenced nutrient sensing and metabolic genes, especially *ilp1*, *ilp2*, *tor*, and *vg*. The elevated expression of *ilp1* and *ilp2* in the sugar syrup treatments suggests that readily available carbohydrates rapidly activate insulin/insulin-like growth factor signaling (IIS), a key pathway regulating nutrient sensing, growth, and energy allocation in honey bees [34,35]. These findings indicate that nutrient-sensing pathways respond rapidly to dietary composition, with detectable transcriptional changes occurring after only 5 days of feeding.

In contrast, honey-based diets and the Cont treatment exhibited transcriptional profiles that were more similar to one another than to sugar syrup alone. This pattern suggests that the greater biochemical complexity of honey and bee bread, including pollen-derived nutrients and other bioactive compounds, may contribute to more balanced metabolic regulation than carbohydrate feeding alone [36,37].

Interestingly, Shoney clustered more closely with Ahoney than with sugar syrup. This finding suggests that biochemical processing during honey ripening may partially modify the nutritional properties of sugar syrup through bee-derived enzymes and metabolites [38,39]. Although sugar syrup-derived honey contains fewer plant-derived phytochemicals and generally exhibits lower biological activity than nectar-derived honey [40], bee-mediated processing may partially compensate for these differences, resulting in transcriptional responses more similar to those induced by natural honey.

Among the pollen-substitute diets, Diet2(M) exhibited the greatest transcriptional similarity to natural bee bread. This observation is consistent with our previous study demonstrating improved nutrient digestibility and honey bee health following supplementation with this probiotic-enriched formulation [28]. Nevertheless, the remaining separation between Diet2(M) and bee bread suggests that artificial pollen substitutes have not yet fully reproduced the biochemical complexity and functional properties of natural pollen resources [18,37].

### 4.2. Diet-Associated Transcriptional Patterns Under Heat-Stress Conditions

Following acute heat stress, dietary groups exhibited distinct transcriptional patterns. Dietary separation was primarily associated with genes involved in nutrient sensing, stress responses, and immune regulation. This observation is consistent with recent studies showing that thermal stress induces coordinated regulation of metabolic, endocrine, and immune pathways rather than independent activation of individual stress responses [37,41].

The loading analysis identified *tor*, *AmGr10*, *ilp2*, *jhamt*, and *hsp70* as the major contributors to dietary separation under heat stress, suggesting coordinated regulation between nutrient-sensing pathways and cellular stress responses. The TOR pathway integrates nutritional status with growth and stress adaptation, whereas HSP70 protects cellular proteins from heat-induced damage and maintains proteostasis during thermal challenge [42,43,44]. Together, these findings indicate that metabolic gene expression differed among dietary treatments under acute heat-stress conditions.

The contribution of *AmGr10* is also noteworthy. Although originally identified as a gustatory receptor, increasing evidence indicates that *AmGr10* participates in nutritional regulation and may contribute to thermal adaptation [29,45]. A recent transcriptomic study further linked *AmGr10*-associated regulation to nutritional performance under temperature stress [46], supporting a broader physiological role for this receptor during thermal challenge.

Honey-fed bees exhibited transcriptional profiles distinct from those of sugar syrup-fed honey bees under heat stress. Although both provide similar carbohydrate resources, honey contains diverse bioactive compounds that contribute to antioxidant capacity and metabolic homeostasis [36,47]. Likewise, bee bread and the Cont treatment also differed from sugar syrup, highlighting the importance of pollen-derived nutrients in maintaining transcriptional regulation during thermal challenge [36,48].

Under heat-stress conditions, the three pollen substitute diets showed broadly similar transcriptional profiles, whereas immune-related transcriptional profiles exhibited less separation between carbohydrate- and protein-based diets. Nevertheless, diet-dependent differences remained evident, particularly for several metabolic and stress-related genes.

In contrast, sugar syrup feeding was associated with higher expression of immune-related genes, particularly *relish* and *defensin2*. Similar transcriptional responses have been reported during pathogen infection, pesticide exposure, and *Varroa destructor* infestation, where immune activation reflects physiological stress rather than enhanced immune competence [25,49]. Therefore, the elevated immune-related gene expression observed in sugar syrup-fed bees likely represents stress-associated immune activation resulting from nutritional imbalance rather than enhanced immune protection.

### 4.3. Diet-Associated Metabolic and Immune Gene Expression Patterns Revealed by Discriminant Analysis

Linear discriminant analysis (LDA) provided complementary insight into the transcriptional responses by identifying the combinations of genes that best distinguished dietary groups under baseline and heat-stress conditions. Unlike PCA, which described the overall relationships among dietary treatments, LDA highlighted coordinated transcriptional signatures associated with different nutritional backgrounds, suggesting that dietary differences were associated with coordinated expression of multiple metabolic and immune-related genes rather than isolated changes in individual genes.

An important finding of the LDA was that dietary discrimination was characterized by distinct transcriptional signatures under baseline and heat-stress conditions. This observation indicates that different genes contributed to dietary discrimination in the separate baseline and heat-stress analyses. Such coordinated regulation is consistent with the complex interactions among nutritional, endocrine, and stress-responsive pathways reported in honey bees [34,41,42].

The LDA also showed that dietary groups remained distinguishable under heat stress despite reduced separation of immune-related transcriptional profiles. This finding indicates that diet-associated transcriptional differences remained detectable under acute heat-stress conditions. Together with the PCA results, these findings indicate that dietary background is associated with coordinated expression patterns across multiple genes rather than isolated changes in individual genes [37].

### 4.4. Implications for Honey Bee Management Under Climate Change

From an applied perspective, our findings suggest that supplemental feeding strategies are associated with distinct transcriptional profiles under both baseline and acute heat-stress conditions. Compared with sugar syrup alone, nutritionally complex diets were associated with distinct metabolic and immune-related transcriptional profiles under thermal challenge.

Although sugar syrup remains a widely used carbohydrate source for colony management, particularly before overwintering [50], sugar syrup feeding was associated with higher expression of selected immune-related genes under heat-stress conditions. In contrast, protein-containing diets, including bee bread and the experimental pollen substitutes, exhibited distinct transcriptional profiles related to nutrient regulation under heat-stress conditions.

As climate change is expected to increase the frequency and intensity of extreme temperature events, improving the nutritional quality of supplemental diets may represent a practical strategy to support honey bee health under changing climatic conditions and sustain pollination services [41]. However, the present study was conducted under controlled cage conditions and focused on short-term transcriptional responses to acute heat stress. Food intake and survival were not evaluated together with gene expression, preventing assessment of the relationship between dietary consumption, molecular responses, and colony performance. Future studies integrating these parameters under field conditions will provide a more comprehensive understanding of how nutrition influences honey bee health under environmental stress.

## 5. Conclusions

This study demonstrates that dietary quality is associated with distinct transcriptional profiles under acute heat-stress conditions in honey bees. Under baseline conditions, dietary differences were primarily associated with nutrient-sensing, endocrine, and metabolic gene expression, and distinct transcriptional profiles were also observed following heat stress. Multivariate and discriminant analyses consistently identified metabolic genes as major contributors to the transcriptional differentiation among dietary treatments. Compared with nutritionally complex diets, carbohydrate-based diets were associated with higher expression of several immune-related genes following heat exposure, indicating that dietary differences remained evident under thermal stress. These findings also highlight the importance of considering nutritional status when evaluating honey bee physiology under multiple environmental stressors and changing climatic conditions. Future studies integrating molecular, physiological, and colony-level measurements under field conditions will further clarify the role of nutrition in supporting honey bee health.

## Data Availability

The data presented in this study are available from the corresponding author upon reasonable request.

## Supplementary Materials

The following supporting information can be downloaded at: https://www.mdpi.com/article/10.3390/insects17080780/s1, Table S1. Composition (%) of ingredients in pollen substitute diets. Table S2. PCR primer sequences. Table S3. PCA factor loadings (F1) of nutritional and immune-related variables after 5-day feeding and heat-stress treatments. Figure S1. Heatmap showing hierarchical clustering of gene expression profiles in honey bees across dietary treatments under (a) baseline feeding conditions (5 days) and (b) heat-stress conditions.
