# Supplementary material for: Diet Quality Shapes Metabolic and Immune Transcriptional Responses of Honey Bees Under Acute Heat Stress
Source: Insects. 2026 Jul 28;17(8):780. doi: 10.3390/insects17080780 (PMC13513611; doi:10.3390/insects17080780)
Supplement: Supplementary file 1 [file insects-17-00780-s001.zip › insects-4441616-supplementary.pdf]

Table S1. Composition (%) of ingredients in pollen substitute diets.

Ingredients	Diet 1(S)	Diet 2(M)	Diet 3(O)
Brewer's yeast	39.69	39.69	39.69
Egg yolk	2.21	2.21	2.21
Sugar	35.36	35.36	35.36
Boiled water	7.16	7.16	7.16
Canola oil	1.01	1.01	1.01
Cellulose	0.88	0.88	0.88
Wheat bran powder	0.88	0.88	0.88
Multiple vitamins	0.44	0.44	0.44
L-methionine	0.10	0.10	0.10
L-lysine	0.24	0.24	0.24
Citric acid	1.85	1.85	1.85
IMP	0.00	0.00	0.00
GMP	0.00	0.00	0.00
Tangerine juice	4.00	4.00	4.00
Apple juice	4.00	4.00	4.00
Chlorella powder	0.08	0.08	0.08
Soytide powder (S)	2.21	-	-
Microbiome-fermented defatted soybean powder (M)	-	2.21	
Defatted soybean powder (O)	-	-	2.21-

IMP means inosine-5′-monophosphate, and GMP means guanosine-5′-monophosphate. M is a fermented form of defatted soybean flour instead of Soytide.

Table S2. PCR primer sequences.

	Locus	5'-3'	Primers	Amplicon Length (bp)	NCBI Reference Sequence
1	*ilp1*	F	CGA TAG TCC TGG TCG GTT TG	237	XM_026442144.1
2		R	CAA GCT GAG CAT AGC TGC AC		
3	*ilp2*	F	TTC CAG AAA TGG AGA TGG ATG	166	XM_026444890.1
4		R	TAG GAG CGC AAC TCC TCT GT		
5	*tor*	F	AAC AAC TGT TGC TGA CGG TG	153	XM_006566642.3
6		R	GTT GCA GTC CAG GCT TTT TG		
7	*jhamt*	F	TTG GAC ATA GGT TGC GGA CC	302	NM_001327967.1
8		R	AAT CCT TTT CCT CCT GGC CG		
9	*vg*	F	GCA GAA TAC ATG GAC GGT GT	146	NM_001011578.1
10		R	GAA CAG TCT TCG GAA GCT TG		
11	*AmGr10*	F	TCT AGC GAA CGC GAC CGA AA	174	XM_006567110.3
12		R	AGG TTC CAC GCG GAA TCA GT		
13	*relish*	F	TCCATTGCATGCAGCACTTCG	264	XM_026444175.1
14		R	ACACATGCACCAGCTTCAGGA		
15	*defensin2*	F	ACCGCTGCTACCACTACGACA	139	NM_001011638.1
16		R	GCCATTTCTGCAACTACCGCCT		
17	*apid1*	F	TTGTTGTTACCTTTGTAGTCGCGGT	70	NM_001011642.1
18		R	AGGCGCGTAGGTCGAGTAGG		
19	*sod1*	F	GCAGTGTGCGTTCTTCAGGGT	86	NM_001178027.1
20		R	TGACCGGTGACCTTCACGGA		
21	*hap70*	F	ATC AAC CTG GCG TCT TGA TTC	104	NM_001160072.2
22		R	AGG TGC AGG TGG AAT GCC AG		
23	*β-actin*	F	GGATTCCTATGTTGGTGATGAAGCCC	177	NM_001185145.1
24		R	GGTGCCTCAGTAAGAAGTACCGGATG		

Table S3. PCA factor loadings (F1) of nutritional and immune-related variables after 5-day feeding and heat-stress treatments.

Treatments	5 days	Heat stress
Factor loadings	F1	F1
Protein	-0.390	-0.230
Carbohydrate	0.393	**0.838**
ilp1	**0.942**	0.662
ilp2	**0.851**	**0.889**
tor	**0.800**	**0.926**
jhamt	-0.010	**-0.843**
vg	**-0.766**	-0.598
AmGr10	0.579	**0.902**
relish	0.498	0.410
defensin2	0.194	0.392
apid1	-0.693	0.663
sod1	-0.241	0.601
hsp70	0.462	**0.894**

Bold values indicate variables with relatively high factor loadings (|loading| ≥ 0.7), representing major contributors to the principal component (F1).

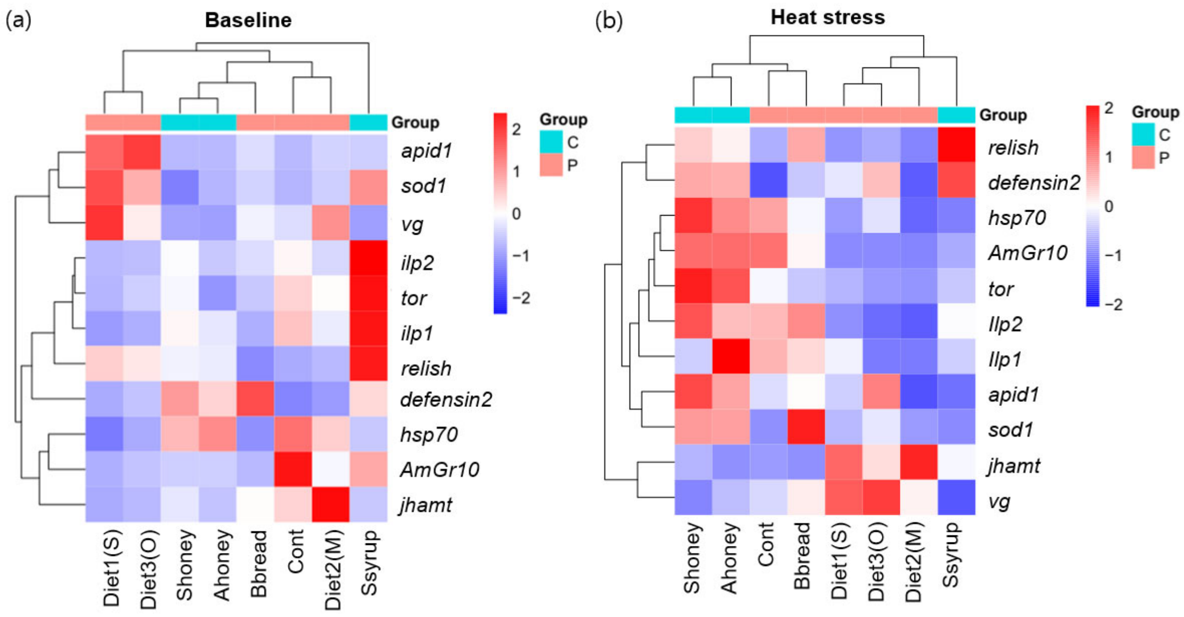

Figure S1. Heatmap showing hierarchical clustering of gene expression profiles in honey bees across dietary treatments under (a) baseline feeding conditions (5 days) and (b) heat-stress conditions. Gene expression levels were normalized and visualized as z-scores, with red indicating relatively higher expression and blue indicating lower expression. Columns represent dietary treatments and rows represent analyzed genes. The dendrograms indicate hierarchical clustering based on similarity in transcriptional profiles among diets and genes. Dietary groups are indicated above the heatmap, where C denotes carbohydrate-based diets, and P denotes protein-containing diets.
